# Phylogenomic Analysis of the Plastid Genome of the Peruvian Purple Maize *Zea mays* subsp. *mays* cv. ‘INIA 601’

**DOI:** 10.3390/plants11202727

**Published:** 2022-10-15

**Authors:** Juan D. Montenegro, Irene Julca, Lenin D. Chumbe-Nolasco, Lila M. Rodríguez-Pérez, Ricardo Sevilla Panizo, Alicia Medina-Hoyos, Dina L. Gutiérrez-Reynoso, Juan Carlos Guerrero-Abad, Carlos A. Amasifuen Guerra, Aura L. García-Serquén

**Affiliations:** 1 Laboratorio de Biología Molecular y Genómica, Dirección de Recursos Genéticos y Biotecnología, Instituto Nacional de Innovación Agraria (INIA), Av. La Molina 1981, Lima 15024, Peru; 2 Department of Neurosciences and Developmental Biology, University of Vienna, Campus-Vienna-Biocenter 1, 1030 Vienna, Austria; 3 School of Biological Sciences, Nanyang Technological University, 60 Nanyang Drive, Singapore 637551, Singapore; 4 Departamento de Fitotecnia, Facultad de Agronomía, Universidad Nacional Agraria La Molina, Av. La Molina s/n, Lima 15024, Peru; 5 Estación Experimental Agraria “Baños del Inca”, Instituto Nacional de Innovación Agraria (INIA), Km. 5.5 Carretera Cajamarca–Celendín, Cajamarca 06000, Peru

**Keywords:** purple maize, *Zea mays* subsp. *mays* cv. ‘INIA 601’, plastid genome, plastid markers

## Abstract

Peru is an important center of diversity for maize; its different cultivars have been adapted to distinct altitudes and water availability and possess an array of kernel colors (red, blue, and purple), which are highly appreciated by local populations. Specifically, Peruvian purple maize is a collection of native landraces selected and maintained by indigenous cultures due to its intense purple color in the seed, bract, and cob. This color is produced by anthocyanin pigments, which have gained interest due to their potential use in the food, agriculture, and pharmaceutical industry. It is generally accepted that the Peruvian purple maize originated from a single ancestral landrace ‘Kculli’, but it is not well understood. To study the origin of the Peruvian purple maize, we assembled the plastid genomes of the new cultivar ‘INIA 601’ with a high concentration of anthocyanins, comparing them with 27 cultivars/landraces of South America, 9 *Z*. *mays* subsp. *parviglumis*, and 5 partial genomes of *Z*. *mays* subsp. *mexicana*. Using these genomes, plus four other maize genomes and two outgroups from the NCBI database, we reconstructed the phylogenetic relationship of *Z*. *mays*. Our results suggest a polyphyletic origin of purple maize in South America and agree with a complex scenario of domestication with recurrent gene flow from wild relatives. Additionally, we identify 18 plastid positions that can be used as high-confidence genetic markers for further studies. Altogether, these plastid genomes constitute a valuable resource to study the evolution and domestication of *Z*. *mays* in South America.

## 1. Introduction

Peruvian agrobiodiversity is well recognized as a source of globally important crops for food and agriculture, such as potato, quinoa, maize, and cotton, among others; and the traditional practices applied by farmers are known to favor the conservation of genetic resources [1]. Among these crops, maize (*Zea mays* L. subsp. *mays*) has been particularly important in the Peruvian diet [2]. To date, 260 maize races have been reported to be indigenous to the American continent [3], and 131 of these are found in the Andean region [4]. In Peru, the first assessment of maize in 1961 identified 49 races [5], and more recently, a new study conducted by specialists on Peruvian maize identified 52 races [6]. Peruvian purple maize is a collection of native landraces selected and maintained by indigenous cultures due to its intense purple color in the seed, bract, and cob. This color is produced by anthocyanin pigments, which have gained interest for their potential use in the food, agriculture, and pharmaceutical industry [7,8].

Despite their phenotypic diversity, studies based on morphological typing and archaeological evidence suggest that all purple maize landraces in Peru originated from the ancient race called ‘Kculli’ [5], or ‘Kulli sara’, which has been cultivated in Peru since prehispanic times [9]. Currently, there are six widely used landraces of purple maize (Morado de Cusco, Morado Canteño, Morado de Caraz, Morado Arequipeño, Negro de Junín, and Huancavelicano) and five improved cultivars (‘INIA-615 Negro Canaán’, ‘INIA-601’, ‘PM-581’, ‘PM-582’, and ‘UNC-47’) [10,11].

Purple maize cultivars have been developed by breeding programs since 1990 [10]. Notably, purple maize is an obligated allogamous plant, and its phenotype can be lost by outcrossing with other non-purple individuals. These characteristics make breeding and germplasm conservation particularly difficult and contribute to the idea that all purple maize individuals share a common ancestor. Due to the effort of the ‘Maize National Program’ conducted by the ‘Instituto Nacional de Innovación Agraria’ (INIA), it was possible to develop a new cultivar designated as ‘INIA 601’ (*Zea mays* subsp. *mays* cv. ‘INIA 601’). This cultivar was bred from two purple maize landraces, ‘Morado de Caraz’ and ‘Negro de Parubamba’, by the recurrent selection of half siblings over six generations. The selection criteria were based on the anthocyanin concentration, total dry mass, and total yield. A recent comparison between improved cultivars and landraces showed that cv. ‘INIA 601’ produces higher yields and higher concentration of anthocyanins than other improved purple maize landraces and cultivars [10,11].

Plastid genes and genomes have been widely used in several species to resolve phylogenetic relationships among different taxa [12,13,14,15,16,17,18]. Some maize plastid genomes are publicly available, including one partial ancient genome (SM10) [19], four *Zea* species, the subspecie *Zea mays* subsp. *huehuetenangensis* (Iltis & Doebley) Doebley, 1990 [20], and several domesticated maize lines including ‘B73’ [16,17,18,19,20,21], ‘B37’ (C, T, S, N), ‘A188’ [22], and ‘Zhengdan958’ [23]. Additionally, the plastid genomes of the close relatives *Tripsacum dactyloides* (L.) L [24] and *Sorgum bicolor* (L.) Moench [17] are also available. However, the plastid genomes of other subspecies of *Z*. *mays* are still missing, namely *Zea mays* subsp. *parviglumis* Iltis & Doebley, 1980 and *Zea mays* subsp. *mexicana* (Schrad.) Iltis, 1971, both of which have contributed enormously to the extant diverse maize germplasm through hybridization and recombination [25]. Furthermore, no plastid genome sequence is available for any South American maize, nor for any purple maize. To answer this, we sequenced and assembled the plastid genome of the cv. ‘INIA 601’ and used public data to assemble the plastid genomes of 27 other cultivars/landraces of South America, 9 *Z*. *mays* subsp. *parviglumis*, and 5 *Z*. *mays* subsp. *mexicana*. The parental individuals of cv. ‘INIA 601’ date back to 1990 [10] and were not available in the Maize Breeding Program. Therefore, these materials could not be included in the phylogenomic analysis. Using these resources, we show that purple maize has multiple origins and that cv. ‘INIA 601’ in particular came from a lineage similar to the landrace ‘Iqueño’. These results also support the hypothesis of a stratified domestication of maize and highlight the need for further molecular studies to fully understand the origin of these landraces.

## 2. Results

### 2.1. Assembly and Identification of Heteroplasmy of cv. ‘INIA 601’ Plastid Genome

**Figure 1:**
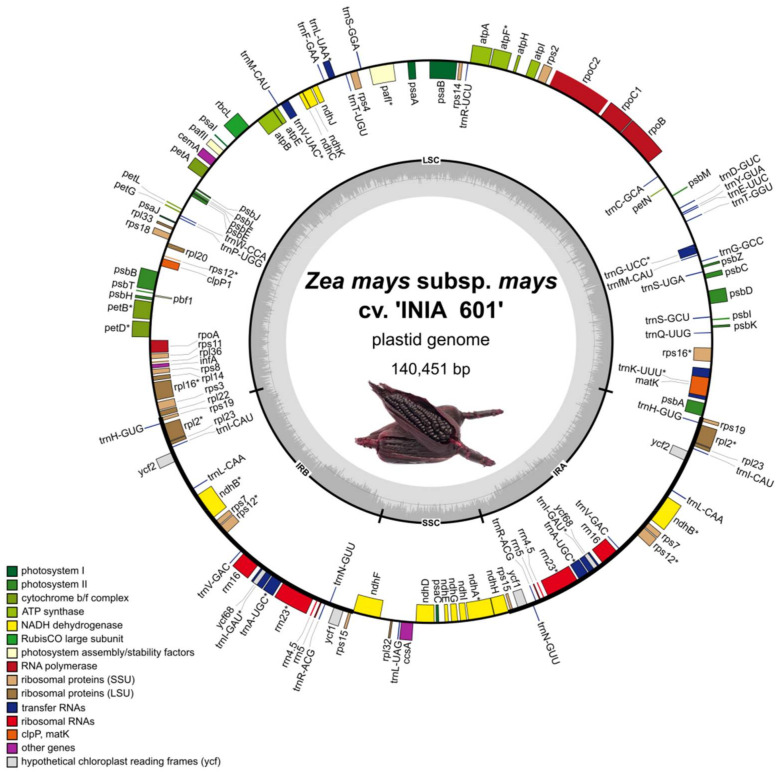
Plastid genome of *Zea mays* subsp. *mays* cv. ‘INIA 601’. The thick lines indicate the IR1 and IR2 regions, which separate the SSC and LSC regions. Genes inside the circle are transcribed in the clockwise direction and genes outside the circle in the counterclockwise direction. Colors of genes indicate their function as shown in the legend. Genes containing introns are marked with an asterisk (*).

To assemble the plastid genome of the cv. ‘INIA 601’, we used PacBio long reads. The initial assembly produced the expected plastid genome size (140 kbp) separated into two contigs. One contig (82,402 bp) contained the long single copy region (LSC), and the other contig (57,988 bp) was formed by the small single copy region flanked by both inverted repeats (SSC, IRA, and IRB). Close inspection of the assembly graph showed that both contigs were constructed out of three connected edges in the graph, typical of a circular molecule with an unresolved repeat. While both single copy (SSC and LSC) edges had an approximate depth of ~20×, the inverted repeat (IR) edge had nearly twice the depth (38×), which is consistent with a collapsed duplicated sequence. To reconstruct the complete circular plastid genome, raw reads were aligned with the assembly graph. Of these, 20 reads spanned the IR edge, 9 of which supported the canonical structural configuration (<IR2<LSC>IR1>SSC), and 11 reads supported the non-canonical structural configuration (<IR2>LSC>IR1>SSC). Upon separation of isoform specific reads, two sets of reads were assembled and resulted in correctly circularized assemblies of a similar size, but with different orientations of their LSC and SSC. The non-canonical isoform was ~10 bp shorter than its canonical counterpart, but both isoforms contained the same number of genes (89 protein coding genes, 38 tRNA, and 8 rRNA) (Figure 1).

### 2.2. Comparison of the Plastid Genomes of Cultivated Individuals and the Teosintes

To have a complete picture of the structural variation among the cv. ‘INIA 601’, the cultivated individuals (*Z*. *mays* subsp. *mays*) and the teosintes (*Z*. *mays* subsp. *parviglumis*, *Z*. *mays* subsp. *mexicana*, *Z*. *mays* subsp. *huehuetenangensis*), we used a total of 46 plastid genomes, from which we assembled the complete genomes of 27 *Z*. *mays* subsp. *mays* and 9 *Z*. *mays* subsp. *parviglumis*, and partial genomes of 5 *Z*. *mays* subsp. *mexicana*; additionally, we recovered four other genomes of *Z. mays* subsp. *mays* from the NCBI database (see Materials and Methods). The plastid genome sizes vary between 140,369 bp (*Z*. *mays* subsp. *parviglumis*—PC_I53_ID1_1) and 140,539 (*Z*. *mays* subsp. *mexicana*—RIMME0026) (Supplementary Table S1). Interestingly, the genomes of *Z*. *mays* subsp. *mexicana* were the largest but could not be circularized. For all the genomes assembled in this project, we annotated a total of 133 genes, of which 87 are protein-coding genes, 38 are transfer RNAs, and 8 are ribosomal RNAs (see Supplementary Table S1). When we compared the annotation of these genomes with the publicly available genomes, we observed a difference in the number of genes annotated. For instance, cv. ‘B73’ and ‘A188’ have 129 genes annotated, cv. ‘Zhengdan958’ has 130, and *Z*. *mays* subsp. *huehuetenangesis* has 131. However, the genomic matching sequences of the missing genes were found using BLAST searches [26] (Supplementary Table S2). Therefore, these differences are explained by the distinct genome annotation methods used.

**Figure 2:**
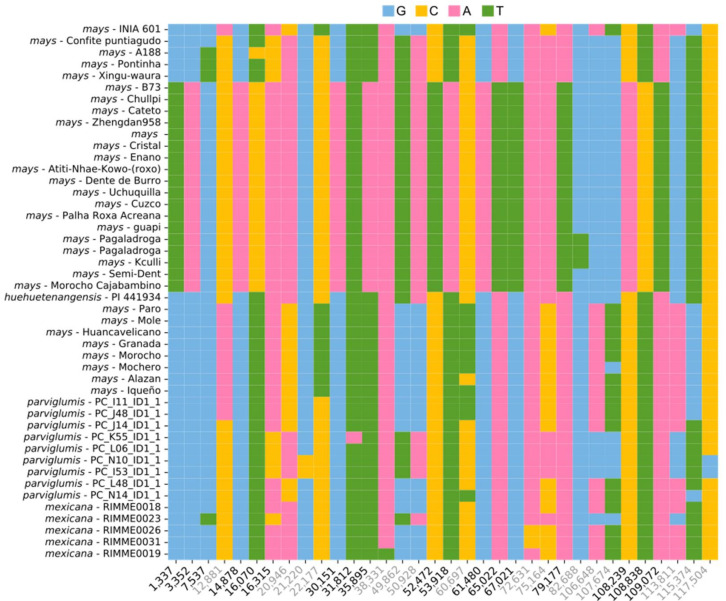
Plot showing the alignment position and identity of plastid SNVs. The 18 high-confidence positions are shown in black, and the other positions are shown in grey. Colors indicate the allele: G—blue, C—yellow, A—pink, T—green.

In order to analyze the sequence variation among the 46 *Z*. *mays* individuals, we aligned the full genomes’ sequences using MAFFT v7.453 [27]. The resulting alignment has 140,647 positions, including 97 SNVs and 445 indels (Supplementary Table S3). When we compare the 97 SNVs to those reported in two other studies, one focused on the analysis of the maize cytoplasmic male sterility [22] and the other one on the plastid genomes of South American maize landraces [28], we observe that 19 and 33 positions are identical in both studies, respectively. Moreover, 18 positions are shared with both studies, which can be used as high-confidence markers (Figure 2, Supplementary Table S3). Our study reports more SNVs’ positions because we did not filter the positions with a low frequency (i.e., positions where one individual has a different allele); therefore, further analyses are needed for confirmation.

### 2.3. Phylogenetic Analysis 

**Figure 3:**
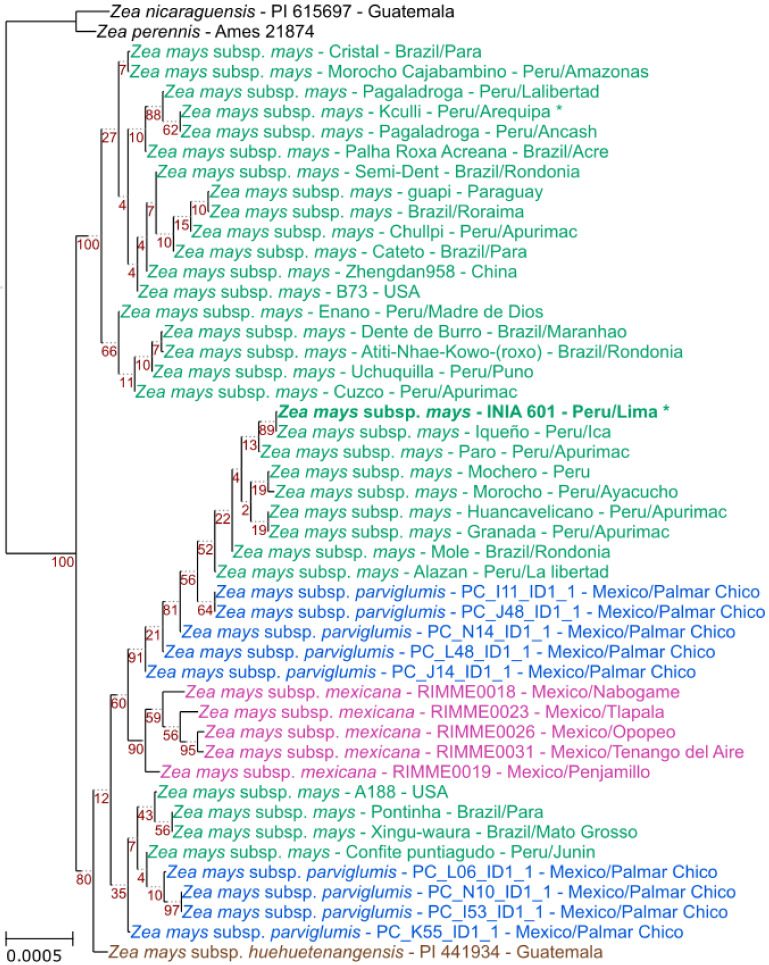
Maximum likelihood species tree. The subspecies of *Zea mays* are shown with different colors: green—*Z*. *mays* subsp. *mays*, blue—*Z*. *mays* subsp. *parviglumis*, pink—*Z*. *mays* subsp. *mexicana*, brown—*Z*. *mays* subsp. *huehuetenangensis*. All bootstrap values are shown in red. The asterisk (*) indicates purple maize.

To understand the evolutionary history of the Peruvian purple maize cv. ‘INIA 601’, we analyzed the 46 plastid genomes used in the previous section plus two outgroups (*Zea nicaraguensis* Iltis & B.F.Benz, 2000 and *Zea perennis* (Hitchc.) Reeves & Mangelsd., 1942). In this phylogeny (Figure 3), we can observe a division of the *Z*. *mays* individuals into two groups. The first group includes the plastid reference genome of the cv. ‘B73’, the hybrid cultivar ‘Zhengdan958’, and other cultivated individuals from Peru, Brazil, and Paraguay. The second group includes all the teosintes (*Z*. *mays* subsp. *parviglumis*, *Z*. *mays* subsp. *mexicana*, and *Z*. *mays* subsp. *huehuetenangensis*) and the fertile inbred lines A188, and other cultivated individuals from Peru and Brazil. Moreover, we can observe that the purple maize individuals do not cluster together, the landrace ‘Kculli’ belongs to the first group, while cv. ‘INIA 601’ belongs to the second group. The cv. ‘INIA 601’ is the sister lineage of the landrace ‘Iqueño’ in the group that includes the other teosintes. These results indicate a polyphyletic origin of the purple maize. The second group of *Z*. *mays* (Figure 3) highlights the geographic movement of plants carrying an ancestral plastid genome more similar to the teosintes. Altogether, these results support the hypothesis of the stratified domestication of maize and the dispersion of a semi-domesticated lineage in South America [29], which was not in contact with other wild populations and conserved the ancestral plastid lineage.

## 3. Discussion

### 3.1. Two Chloroplast Structural Isoforms Coexist in ‘INIA 601’

The plastid genome of cv. ‘INIA 601’ was assembled using long reads, which allowed us to separate two structural paths based on the relative orientation of LSC and SSC alone. All reads that linked the LSC and the SSC in the same direction (>> or <<) were separated from the reads that linked them in opposite directions (< > or > <). The removal of either set from the assembly dataset resulted in a single circular contig with different LSC and SSC orientations. These results suggest that the fragmentation of plastid genome assemblies using long reads is caused by the inability of the assembler to separate conflicting reads that support different co-existing structural configurations of the genome. However, using assembly graphs and graph mapping, we accurately separated reads supporting different structural variations and reconstructed the two isoforms separately. Structural heteroplasmy has been previously reported in both land plants [30] and algae [31]. A recent study of a large sample of land plants has shown that structural heteroplasmy is widespread and that long-read sequences can be used to reconstruct plastid isoforms [32,33].

In maize, an early study of cytoplasmic male sterility used restriction patterns of the cosmid library to demonstrate that recombination between the inverted repeats caused the inversion of the SSC region and, consequently, the co-existence of two structural isoforms [22]. Another study, using nanopore reads, reconstructed two plastid isoforms for *Z*. *mays* subsp. *mexicana* ‘TIL11’, a teosinte inbred line [32]. In our research, the ratio of reads supporting different isoforms was close to 1:1, suggesting that rapid flip-flop recombination shortly after plastome duplication may be responsible for the generation of both structural isoforms, as suggested by previous studies [34,35]. Furthermore, the existence of only two structural conformations, as opposed to the 256 that are theoretically possible, suggests that the recombination happens in a non-random way [32].

### 3.2. Phylogenetic Relationship of Zea Mays Subspecies and Origin of cv. ‘INIA 601’

Previous studies using nuclear and plastid genetic markers support a monophyletic origin of *Z*. *mays* and two genetic groups for South American landraces [28,36,37]. However, the relationship between teosintes and cultivated maize is still discussed [29,38]. For instance, a recent study using plastid SNPs has shown that the teosintes are distributed across all the *Z*. *mays* clusters [28]. Nevertheless, our results using the plastid genomes of 46 individuals show a different pattern and divide the *Z*. *mays* individuals into two groups: I) a group formed by some *Z*. *mays* subsp. *mays*, and II) a second group that clusters the four subspecies (*Z*. *mays* subsp. *mays*, *Z*. *mays* subsp. *parviglumis, Z*. *mays* subsp. *mexicana*, *Z*. *mays* subsp. *huehuetenangensis*). This division agrees with the hypothesis that two waves of semi-domesticated maize dispersed southward out of the center of domestication [29,39]. Moreover, the second group observed in our analysis suggests gene flow between the teosintes and cultivated maize. This group agrees with the Pan-American cluster described previously, where an excess gene flow with teosintes (*Z*. *mays* subsp. *parviglumis*) was reported [29]. Additionally, gene flows between *Z. mays* subsp. *mexicana* and maize have been observed [38]. In summary, these results suggest a complex scenario of maize domestication with recurrent gene flow from wild relatives.

A single origin of the Peruvian purple maize has been proposed [5]. In particular, it has been suggested that this purple maize originated from a single ancestral landrace ‘Kculli’, which appeared in the archaeological registry ~2500 YBP as the proto-Kculli complex. The specific combination of alleles needed for the stable production and accumulation of anthocyanin in seeds and cobs has also been used as evidence that all extant purple maize landraces in South America have a common ancestor. If true, the plastid genome assembled in this study would represent a common conserved sequence shared by most South American purple maize races. However, our results show that cv. ‘INIA 601’ is more closely related to landrace ‘Iqueño’, while the landrace ‘Kculli’ belongs to a different cluster pointing to a polyphyletic origin of purple maize. Nevertheless, a comprehensive phylogenetic study of diverse South American purple maize germplasms is still needed to fully test this hypothesis.

## 4. Materials and Methods

### 4.1. Plant Material

Certified seeds from the cv. ‘INIA 601’ were provided by the Maize Breeding Program from the ‘Instituto Nacional de Innovación Agraria’ (INIA-Peru). Seeds were germinated in vitro, and plantlets were transferred to pots with substrate and grown under greenhouse conditions (22 °C, 70% HR).

### 4.2. DNA Extraction and Sequencing 

Leaves from a single 25-days-old plant were collected, and DNA was extracted following a previously described method [40] with minor modifications. Briefly, 100 mg of leaf tissue was snap-frozen with liquid nitrogen and grounded with a mortar and pestle. The ground material was mixed with 1 mL of 3% CTAB extraction buffer and 1 µL of 2-β-mercaptoethanol and incubated for 60 min at 65 °C. The working solution was mixed by inversion every 10 min. After cooling at room temperature, 2 rounds of cleaning with 700 µL chloroform-isoamyl alcohol (24:1 *v*/*v*) were performed. Approximately 400 µL of supernatant were recovered, and DNA was precipitated with cold isopropanol. Additional washes with 70% ethanol were done after DNA pellet recovery. DNA purity was evaluated using the spectrophotometer Nanodrop 8000 (Thermo Fisher Scientific, Waltham, MA, USA), and the concentration of dsDNA was determined using a QubitTM Fluorometer dsDNA BR Assay Kit (Thermo Fisher Scientific). DNA integrity was examined by agarose gel electrophoresis. Afterwards, DNA integrity was also estimated using a Femto Pulsed field gel electrophoresis (Agilent) at the University of Washington, and the adequate fraction was selected for downstream sequencing on the Pacific Biosciences Sequel II platform using the Blue pippin DNA size selection system. One lane in 1 SMRT cell was run in CLR mode, and 15 h movies were produced.

### 4.3. Genome Assembly and Annotation

#### 4.3.1. Genome Assembly

##### Peruvian Purple Maize (‘INIA 601’)

Long reads were mapped to the reference maize plastid genome (‘B73’, NCBI accession: KF241981) using minimap2 v2.24 [41] with standard parameters. Reads with a mapping quality equal to or above 60 were extracted and assembled with Flye v2.7 with three rounds of polishing [42]. The assembly graph was visualized with Bandage v0.8.1 [43] to identify the edges corresponding to SSC, LSC, and IR. Raw reads were aligned to the assembly graph with Minigraph v0.1 [44] using standard parameters. Reads spanning an IR region were classified into two mutually exclusive conformations: <IR>LSC>IR>SSC and <IR<LSC>IR>SSC. All reads aligned to plastid edges were extracted and separated into two conformation read sets based on the previous classification. A de novo assembly of each set was performed with Flye v2.7 using standard parameters, followed by five iterations of polishing.

##### Zea Mays Subsp. Parviglumis and Zea Mays Subsp. Mexicana

Paired-end Illumina reads were downloaded from NCBI SRA (all accessions are listed in Supplementary Table S3). Plastid genomes were assembled with GetOrganelle v1.7.5.3 [45] using standard parameters and the cv. ‘B73’ reference plastid genome as the anchor. Final circular assemblies were aligned to the cv. ‘B73’ reference using Mummer v4.0 [46] and rotated as needed for multiple sequence alignment.

#### 4.3.2. Annotation

All the genomes were annotated using GeSeq [47] and tRNAscan-SE 2.0 [48]. The circular genome of cv. ‘INIA 601’ was drawn with OGDRAW v1.3.1 [49]. Finally, all plastid genome assemblies and annotations were uploaded to NCBI’s NT (nucleotide) database. Accession numbers are available in Supplementary Table S1.

### 4.4. Phylogenetic Reconstruction

To reconstruct the phylogenetic relationship of *Z. mays*, we used 42 genomes assembled in this project (28 *Z. mays* subsp. *mays*, 9 *Z. mays* subsp. *parviglumis,* and 5 *Z. mays* subsp. *mexicana*) and 4 genomes obtained from the NCBI database (3 *Z. mays* subsp. *mays* and 1 *Z. mays* subsp. *huehuetenangensis*) (See Supplementary Table S1). Additionally, we used *Z*. *nicaraguensis* and *Z*. *perennis* as outgroups. All genomes were aligned using MAFFT v7.453 [27], and the phylogenetic tree was reconstructed using RAxML v8.2.12 [50] with the GTR model. Support values were calculated using 5000 bootstrap searches with the rapid bootstrapping implemented in RAxML. The tree was plotted using ete3 [51], and the alignment plot was generated with the seaborn python package [52].

## 5. Conclusions

In this paper, we report the first complete plastid genome of a purple maize and use publicly available data to reconstruct additional plastid genomes for *Z*. *mays* subsp. *parviglumis*, *Z*. *mays* subsp. *mexicana*, and other South and North American maize landraces. We used these genomes to assess the origin and domestication of purple maize. Our results suggest a polyphyletic origin of purple maize in South America and agree with a complex scenario of domestication with recurrent gene flow from wild relatives. We also identify 18 plastid positions that can be used as high-confidence genetic markers.

## Data Availability

The data is deposited at NCBI (see the Supplementary Table S1).

## Supplementary Materials

The following supporting information can be downloaded at: https://www.mdpi.com/article/10.3390/plants11202727/s1, Supplementary Table S1. List of individuals included in this study. The first and second columns indicate the sample name and the cultivar/landrace/race/isolate/ecotype name. The third to sixth columns indicate the geographical origin. The seventh column shows the source of the reads and the last column the reference of the paper. Supplementary Table S2. Blast results of the non-annotated genes. Supplementary Table S3. Variable positions in the alignment of the 46 plastid genomes. In the first row, the positions in the alignment are shown. In the following rows, the genomic positions of the sample and the nucleotide are shown. The asterisk (*) and the plus sign (+) mark the positions also reported in the work of Bosacchi et al. (2015) [22] and López et al. (2021) [28], respectively.
